# Type VI secretion systems in plant‐associated bacteria

**DOI:** 10.1111/1462-2920.13956

**Published:** 2017-11-10

**Authors:** Patricia Bernal, María A. Llamas, Alain Filloux

**Affiliations:** ^1^ Department of Life Sciences, MRC Centre for Molecular Bacteriology and Infection, Flowers Building, 1st floor South Kensington Campus Imperial College London London SW7 2AZ UK; ^2^ Department of Environmental Protection Estación Experimental del Zaidín‐Consejo Superior de Investigaciones Científicas 18008 Granada Spain

## Abstract

The type VI secretion system (T6SS) is a bacterial nanomachine used to inject effectors into prokaryotic or eukaryotic cells and is thus involved in both host manipulation and interbacterial competition. The T6SS is widespread among Gram‐negative bacteria, mostly within the Proteobacterium Phylum. This secretion system is commonly found in commensal and pathogenic plant‐associated bacteria. Phylogenetic analysis of phytobacterial T6SS clusters shows that they are distributed in the five main clades previously described (group 1–5). The even distribution of the system among commensal and pathogenic phytobacteria suggests that the T6SS provides fitness and colonization advantages *in planta* and that the role of the T6SS is not restricted to virulence. This manuscript reviews the phylogeny and biological roles of the T6SS in plant‐associated bacteria, highlighting a remarkable diversity both in terms of mechanism and function.

## Introduction

**Figure 1:**
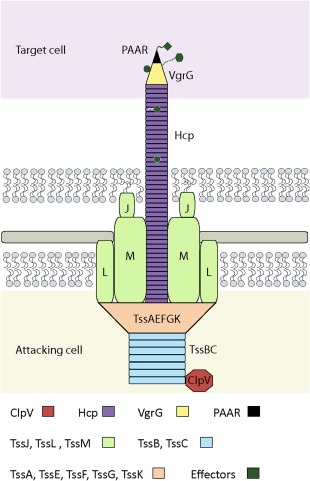
Schematic representation of the T6SS structure. Baseplates components (TssA, TssE, TssF, TssG and TssK are coloured in light orange whereas membrane complex is represented in green (TssJ, TssL and TssM) and the sheath components TssB and TssC in blue.

The bacterial T6SS is a molecular nanoweapon used to inject toxic effectors into eukaryotic and prokaryotic cells (Ho *et al*., 2013). This secretion system can be used by bacteria to manipulate and subvert eukaryotic host cells and/or to fight other bacteria thriving in the same ecological niche (Ma and Mekalanos, 2010; Ho *et al*., 2013). In fact, the microorganisms that possess a T6SS appear to have a significant fitness advantage within a polymicrobial community. T6SS‐active organisms synthesize immunity proteins concomitantly with T6SS toxins to prevent self‐intoxication or being targeted by sister cells. The T6SS toxins are injected into target cells via a supramolecular complex that expands from the cytoplasm to the outer membrane (Fig. 1). This complex is made of thirteen distinct constituents, or core components, named Tss (for Type six secretion), usually all encoded within the same gene cluster (Fig. 1) (Leiman *et al*., 2009; Felisberto‐Rodrigues *et al*., 2011; Kapitein *et al*., 2013; Shneider *et al*., 2013; Silverman *et al*., 2013; Kudryashev *et al*., 2015; Cianfanelli *et al*., 2016; Planamente *et al*., 2016; Taylor *et al*., 2016). Besides, most T6SS clusters encode additional proteins known as accessory components (generally quoted Tag from Type VI accessory genes), which modulate the assembly of the system and/or are involved in their regulation. Structurally the T6SS resembles a phage tail‐like device formed by a rigid tube of hexameric Hcp rings that is wrapped in a contractile sheath (Fig. 1). While the tube is a stack of rings, the sheath is made of two proteins, TssB and TssC, arranged in a helical configuration. The tail polymerization expands in the cytosol and is initiated from a so‐called baseplate structure constituted by the TssA, TssE, TssF, TssG and TssK proteins. The baseplate connects the tail part of the T6SS onto the cytoplasmic membrane by interacting with an integral membrane complex formed by the TssL, TssM and TssJ proteins. This structure connects with the sheath via the baseplate (Fig. 1). The Hcp tube is topped by a torch‐like trimer of VgrG proteins and a PAAR‐sharpening tip (Fig. 1). The inner tube of Hcp components together with the puncturing device made of VgrG and PAAR are propelled outside the cell, and generally into a target cell, on a sheath contraction. Therefore, although Hcp and VgrG proteins are considered structural components of the secretion machine, they are detectable in the extracellular medium of T6SS‐active bacteria, which makes a convenient readout to monitor T6SS activity *in vitro* (Pukatzki *et al*., 2006). The last core component of the T6SS is the ClpV ATPase, which is responsible for disassembling the contracted sheath thus permitting the recycling of its components for subsequent secretion/firing events (Fig. 1).

Many T6SS effectors and their cognate immunity proteins (known as EI pairs) are coded by genes genetically linked to *hcp, vgrG* or *paar* genes (Dong *et al*., 2013; Hachani *et al*., 2014; Ma *et al*., 2014; Liang *et al*., 2015). This genetic association correlates with a specific molecular interaction between the effector and the Hcp or the VgrG/PAAR proteins. As such, the T6SS can deliver effectors into target cells either escorted by the Hcp tube or by association with the VgrG/PAAR tip‐forming complex (Shneider *et al*., 2013; Hachani, *et al*., 2014; Whitney *et al*., 2014). Moreover, the T6SS is quite modular and can accommodate different combinations of VgrG/PAAR proteins to form the tip (Cianfanelli, *et al*., 2016). Altogether this implies that multiple effectors can be delivered at once on a single contraction event and by a single T6SS (Shneider *et al*., 2013; Silverman *et al*., 2013; Hachani *et al*., 2014; Whitney *et al*., 2014).

T6SSs are found in around 25% of Gram‐negative bacteria, mainly within the Proteobacterium phylum and the classes α‐, β‐ and γ‐proteobacteria (Boyer *et al*., 2009), including a large number of phytobacteria. From the appearance of the first eukaryotic cells around two billion years ago (Umen, 2014), bacteria and plants have co‐evolved. Yet, most bacteria associated with plants are commensal and have no apparent beneficial or detrimental effect on the host. These commensal microorganisms can be found anywhere on the plant although most frequently on leaves, roots and fruits, where they generally live epiphytically although in few cases endophytic bacteria are found within the plant vessels (Turner *et al*., 2013). Other plant‐associated microorganisms have developed a beneficial relationship with the plants, such as the so‐called plant growth promoting rhizobacteria (PGPR). These bacteria provide the plant with important services such as nitrogen fixation, solubilization of minerals and synthesis of plant hormones, among others, thus establishing intimate relationship with the plant (e.g., rhizobia microorganisms and leguminous plants) (Turner *et al*., 2013). Some PGPRs are also biocontrol agents and are able to protect plants from the detrimental effect of phytopathogens by competing with the pathogen for an ecological niche or a substrate, producing inhibitory substances, or inducing systemic resistance in host plants to the pathogen (Turner *et al*., 2013). Although only a very small fraction of plant‐related bacteria causes disease, the effects of this small fraction on crop plants have a major impact on agriculture resulting in significant economic losses (Oerke, 2006). Because of this major threat on human resources, plant pathogens are widely studied, and analysis of T6SS in phytobacteria has been initially focussed in this group. The fact that the top ten plant pathogenic bacteria (Mansfield *et al*., 2012), including *Pseudomonas syringae* pathovars and *Ralstonia solanacearum* strains, encode T6SS clusters initially suggested a major role for this secretion system in plant colonization and virulence (Sarris *et al*., 2010). However, recent analyses of the T6SS in non‐pathogenic phytobacteria have shown that the T6SS is associated with important functions that are beyond virulence, as reviewed here.

## T6SS in *Rhizobium*: a prelude to a golden story

The term T6SS was first coined by Pukatzki and colleagues (2006) who discovered the system in the human pathogen *Vibrio cholerae*. However, the genes encoding this secretion system were originally reported in the symbiotic phytobacterium *Rhizobium leguminosarum* (the *imp* genes) (Bladergroen *et al*., 2003). Bacteria of the genera *Rhizobium* can establish symbiosis with leguminous plants in specially developed organs, the root nodules, supplying the plant with fixed nitrogen in exchange for carbon sources (Suzaki and Kawaguchi, 2014). The *Rhizobium*‐legumes symbiosis exhibits high species‐specificity, and successful nodule formation requires the exchange of specific chemical signals (Garcia *et al*., 2015; Nelson and Sadowsky, 2015). These signals include plant‐produced flavonoids that induce the synthesis of the lipo‐chitooligosaccharide (LCO) nodulation (Nod) factor by *Rhizobium*, the final structure of which is species‐specific and only recognized by compatible hosts. *R. leguminosarum* strain RBL5523 is a derivative of *R. leguminosarum* bv. *trifolii* that bears a plasmid encoding the Nod factors from the *R. leguminosarum* bv. *viciae* strain. This plasmid allows RBL5523 to moderately nodulate pea plants, which is not the natural host of this strain, but not to fix nitrogen. A transposon (Tn5) mutant of RBL5523 was able to form nodules in pea much more efficiently than the wild‐type and to fix nitrogen (Bladergroen *et al*., 2003). The transposon insertion was located in a gene cluster that was originally named *imp* (for impaired in nodulation), a nomenclature that has been later changed to *tss* (for type six secretion) once the term T6SS was coined. The Tn5 mutation, which is located in *tssK* (*impJ*) (Fig. 1), led to the absence of at least four proteins in the supernatant of the mutant strain (Bladergroen *et al*., 2003). One of these was proposed to be RbsB, a putative periplasmic ribose binding protein encoded within the *rbsDABCK* ABC transport system and likely involved in the transport of ribose into the cell. However, the predicted role of this protein in the periplasm and the presence of an N‐terminal signal peptide in the precursor form (Bladergroen *et al*., 2003) are no features of T6SS secreted proteins. As such it raises the question whether RbsB is a genuine T6SS substrate or if its presence in the supernantant was the result of cell leakage, a possibility that in fact the authors did not exclude (Bladergroen *et al*., 2003). Nevertheless, addition of a RBL5523 wild‐type culture supernatant to pea plants inoculated with the *tssK* mutant considerably reduced the amount of nodules and nitrogen fixation, while addition of the mutant supernatant did not (Bladergroen *et al*., 2003). This suggests that the inhibition is indeed performed by a T6SS secreted factor(s), which is an important observation since it implicates that RBL5523 T6SS effectors can be directly secreted in the supernatant without being necessarily injected into target cells to produce an effect. Maybe these effectors are taken up *a posteriori* by the host cells, or perhaps they influence the composition of the extracellular environment thus impacting on host cell behaviour. Along these lines, it has been recently shown that *Pseudomonas taiwanensis* is able to secrete a siderophore (i.e., pyoverdine) in a T6SS‐dependent manner (Chen *et al*., 2016) and this iron‐chelating compound is thus not injected into target cells. Similarly, T6SSs in *Yersinia pseudotuberculosis*, *Burkholderia thailandensis* and *Pseudomonas aeruginosa* have been involved in zinc, manganese and iron acquisition respectively (Wang *et al*., 2015; Chen *et al*., 2016; Lin *et al*., 2017; Si *et al*., 2017). These systems represent a potential new category of T6SSs that secrete effectors that are not directly injected into target cells. Although the mechanism by which *R. leguminosarum* RBL5523 T6SS effectors influences nodulation is still unknown, it has been shown that co‐inoculation of RBL5523 wild‐type and *tssK* mutant strains resulted in nodules in which only the mutant was present (Bladergroen *et al*., 2003). Altogether these results suggest that the *R. leguminosarum* RBL5523 T6SS and the T6SS secreted factors impair pea infection and root nodule formation. Microscopic examination of the few nodules formed by the RBL5523 wild‐type strain showed that this strain is unable to infect the plant tissue, a process necessary for efficient nodule formation and the authors propose that it is due to a defence response of the host triggered by T6SS effectors (Bladergroen *et al*., 2003).

The work of Bladergroen and colleagues (2003) described here thus represents the first study of T6SS in bacteria, and was done in a phytobacterium, *Rhizobium leguminosarum*. However, the key breakthrough in T6SS discovery occurred three years later and was described in two animal pathogens, *V. cholerae* and *P. aeruginosa* (Mougous *et al*., 2006; Pukatzki *et al*., 2006). Since then, an increasing number of T6SS studies, mainly in animal pathogens, have revealed many of the mechanistic and functional features of this important secretion system. Although still scarce when compared to studies of T6SS in animal pathogens, the analysis of this system is a topic that is increasingly coming back to the phytobacteria field.

## Phylogenetic analysis of T6SS in plant‐related bacteria

**Figure 2:**
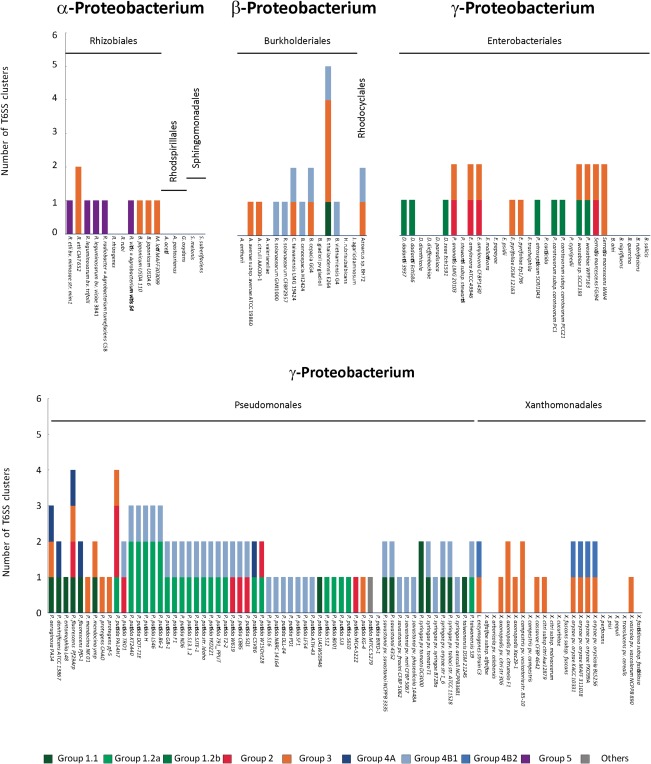
Number of T6SS clusters in plant associated bacteria. Bacteria are distributed in α‐, β‐ and γ‐proteobacteria. T6SS clusters are represented with different colours according to their phylogenetic groups: group 1 (green), group 2 (red), group 3 (orange), group 4 (blue) and group 5 (purple).

**Figure 3:**
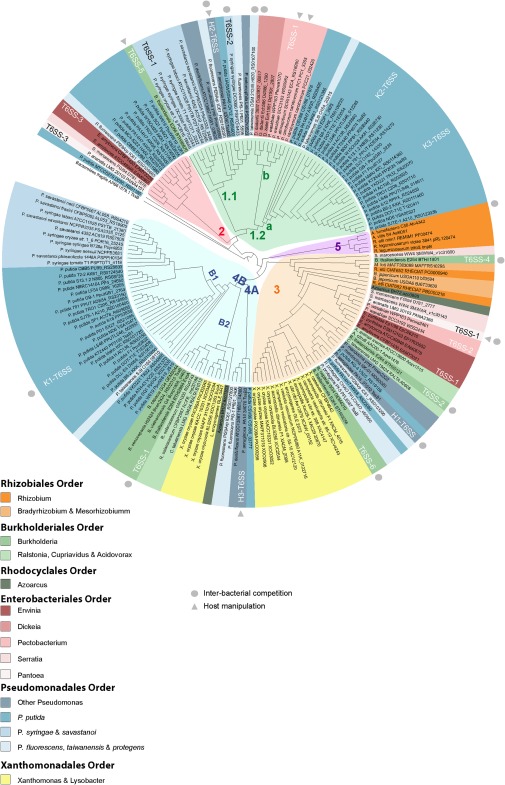
Phylogenetic distribution of T6SS clusters in plant‐associated bacteria. Maximum‐likelihood tree with 1000 bootstrap replicates was built with Mega 6 for the core component protein TssB. T6SS cluster nomenclature (Boyer *et al*., 2009; Barret *et al*., 2011) is used to show the major phylogenetic clusters. Five main groups are clearly distinguishable: group 1 (green), group 2 (red), group 3 (orange), group 4 (blue) and group 5 (purple). Subgroups 1.1 and 1.2a and 1.2b are indicated in the tree (green). Subgroup 4A and 4B1 and 4B2 are represented in blue. A grey circle indicates T6SSs involved in interbacterial competition whereas a grey triangle represents systems involved in host manipulation. Characterized T6SSs with specific name in the literature are indicated in the tree next to the strain name.

We have used a comprehensive list of plant pathogenic and plant‐associated bacteria (Beattie, 2006; Bull *et al*., 2010) to screen genomes for T6SS clusters and get more insights into T6SS in phytobacteria. A total of 143 genomes within the Proteobacterium Phylum, 80 from pathogenic and 63 from beneficial phytobacteria, have been included in this analysis (Fig. 2). We have identified a total of 170 T6SS clusters distributed in 104 strains, including plant pathogens, symbionts and plant‐growth promoting rhizobacteria (Fig. 2). A single strain can contain from 1 to 5 clusters, although only 7% possess more than two (Fig. 2). Phylogenetic analysis shows that these phytobacterial T6SSs are distributed among the five main clades described previously by Boyer and colleagues (2009). Most of them, 124 (87%), belong to groups 1, 3 or 4 (Fig. 3). Strains with more than one T6SS normally contain clusters from different phylogenetic clades (Figs 2 and 3), suggesting that the acquisition of T6SS traits mainly occurs by horizontal gene transfer and not as the result of duplication, although duplication of T6SS clusters has been reported in phytobacteria (Bernal *et al*., 2017). Based on phylogenetic analysis of T6SS in *Pseudomonas* strains, these five initial T6SS clades have been further subdivided in subgroups 1.1 and 1.2 within clade 1, or 4A and 4B within clade 4 (Barret *et al*., 2011). Of note, our analysis shows that all phytobacteria found in group 1.1 are *Pseudomonas* species (Fig. 3). Within group 1.2, two branches can be observed, one containing *Pseudomonas* T6SS clusters (mainly from *P. putida* strains) (Fig. 3, subgroup 1.2a) and another including non‐*Pseudomonas* species from the *Dickeya* and *Pectobacterium* genera (Fig. 3, subgroup 1.2b). Representation of phytobacterial T6SS clusters in subgroup 4A is poor with only five strains (Fig. 3). As observed for group 1.2, group 4B comprises two branches, one specific for *Pseudomonas* species (Fig. 3, subgroup 4B1) and another more diverse with species from the genera *Azoarcus*, *Xanthomonas*, *Ralstonia* and *Burkholderia* (Fig. 3, subgroup 4B2). In group 2, T6SS clusters from a variety of genera including *Pseudomonas*, *Erwinia*, *Serratia* and *Pantoea* are found (Fig. 3). Finally, group 3 is the most heterogeneous clade including strains from practically all genera included in this study (Fig. 3), while group 5 is extremely homogenous containing exclusively species from the *Rhizobium* genus (Fig. 3).

In conclusion, it seems like some T6SS clusters are more prompted to be acquired by horizontal gene transfer and thus are found in a great variety of genera (i.e. group 3). Meanwhile, other clusters are found conserved in specific genera which could indicate that they were acquired from a common ancestral before the different species of the genus diversified (i.e., group 1.1, 4B1).

Overall, the fact that T6SS clusters from the five clades are also present in plant‐related bacteria indicates that evolution of T6SS has been independent of a specific niche or host. Nevertheless, there have been several attempts to correlate T6SS clades with different functions or ecological niches (Boyer *et al*., 2009; Schwarz *et al*., 2010). For example, Boyer and colleagues (2009) referred to group 4 as ‘the plant related group’ since they mainly found this T6SS cluster in plant‐related strains. However, our phylogenetic study suggests that this designation is not appropriate as phytobacterial T6SSs can be found among all clades (Fig. 3). Other studies suggested that clusters involved in different function could branch in different phylogenetic groups (Schwarz *et al*., 2010). For example, *B. thailandensis* E264, a strain that contains five T6SS clusters, seems to have only one system involved in host manipulation (T6SS‐5) (Schwarz *et al*., 2010). This system branches in clade 1, while the other four, which are involved in interbacterial competition, branch in clade 3 and in clade 4 (Figs 2 and 3). Studying the distribution of these clusters together with 300 T6SSs from different species suggested that those involved in interbacterial competition (T6SS‐1, T6SS2, T6SS‐4 and T6SS‐6) were differently distributed comparing to the one involved in host manipulation (T6SS‐5) (Schwarz *et al*., 2010). However, a closer look at other T6SS clusters found in the branch where the *B. thailandensis* T6SS‐5 is (Fig. 3, group 1) shows that many of them are involved in interbacterial competition (Fig. 3, group 1 – grey circles). This observation thus challenges the neat distinction envisioned with the *B. thailandensis* T6SSs and suggests that it cannot be generalized. Furthermore, several T6SSs have been involved both in host manipulation and interbacterial competition (Lesic *et al*., 2009; Sana *et al*., 2012; Russell *et al*., 2013; Jiang *et al*., 2016), thus making the attempt to classify the clusters according to their function even more unrealistic. Instead, it is reasonable to consider that the role of a specific T6SS is primarily driven by the function of the effector(s) that the system secretes. Yet, considering that a particular effector can target both eukaryotic and prokaryotic cells, e.g., Tle4 of *P. aeruginosa* (Russell *et al*., 2013), a T6SS might have a more promiscuous role than previously anticipated. In fact, although several T6SSs from pathogenic phytobacteria were initially described as virulence traits, recent work is showing that T6SS displays minor effects on plant manipulation and are rather involved in interbacterial competition. An example is the T6SS of *Agrobacterium tumefaciens* DC58, which was initially proposed to enable this strain to cause tumors in plants (Wu *et al*., 2008), but which has also been recently described as a system primarily involved in interbacterial competition *in planta* (Ma *et al*., 2014). Similarly, the H2‐ and the H3‐T6SS of the opportunistic pathogen *P. aeruginosa* are involved in both interbacterial competition and host manipulation (Fig. 3) (Lesic *et al*., 2009; Russell *et al*., 2013; Jiang *et al*., 2016, 2014; Allsopp *et al*., 2017).

In conclusion, not only it seems unfeasible to group T6SS clusters by function but even dividing commensal and pathogenic phytobacteria based on T6SS does not seem to be phylogenetically supported. In most clades, co‐occurrence of T6SS clusters from commensal and pathogenic strains is observed, even in clade 5 that contains very few representatives (Fig. 3). In any cases, the wide distribution of T6SS clusters in plant‐associated bacteria suggests that this molecular weapon is important for optimal fitness during plant colonization be it to cope with the plant response as much as with the resident microbiota.

## Genetic organization of phytobacterial T6SS clusters

T6SSs have been divided in four different subtypes due to the presence of atypical T6SS clusters in the *Francisella* genus and the *Bacteroidetes* phylum (Russell *et al*., 2014; Bock *et al*., 2017). The canonical or general T6SS found mainly in Proteobacteria constitutes subtype 1 (T6SS^i^). *Francisella* T6SS forms subtype 2 (T6SS^ii^), the main T6SSs present in *Bacteroidetes* represent subtype 3 (T6SS^iii^) and the recently found in *Amoebophilus asiaticus* is the distant subtype 4 (T6SS^iv^). The homology between the components from T6SS^i^ and T6SS^ii^/T6SS^iii^/T6SS^iv^ is low but, with some exceptions, most T6SS^i^ core components are found in these other systems (Russell *et al*., 2014; Bock *et al*., 2017). For example, the T6SS^ii^ and T6SS^iv^ gene clusters lack homologs of the T6SS^i^ protein ClpV (Bröms *et al*., 2010; Bock *et al*., 2017) whereas homologs of the T6SS^i^ membrane complex (TssL, TssM and TssJ) and the baseplate component TssA (Planamente *et al*., 2016) are absent from T6SS^iii^ and T6SS^iv^ or at least very divergent in terms of primary amino acid sequence (Russell *et al*., 2014; Bock *et al*., 2017; Spidlova and Stulik, 2017). In any case, T6SS^ii^, T6SS^iii^ and T6SS^iv^ are absent in plant‐related bacteria, therefore, only the genetic organization of systems from the T6SS^i^ subtype (referred as T6SS) will be described in this section.

**Figure 4:**
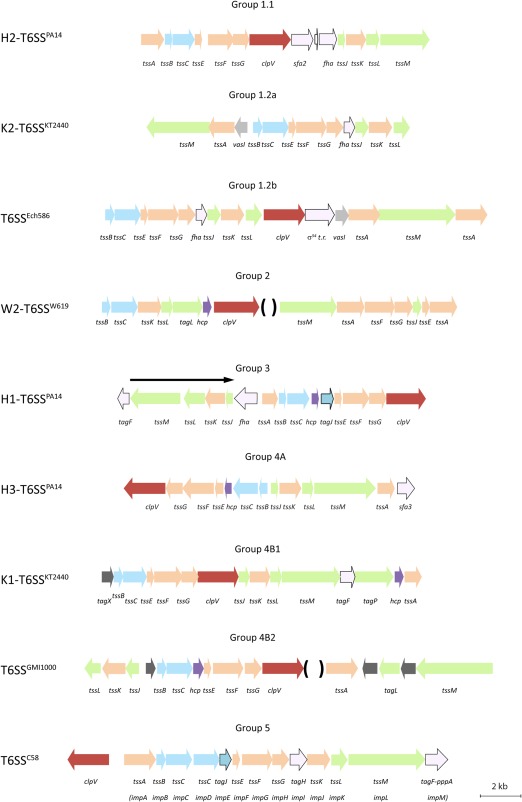
T6SS clusters in *P. putida*. Genetic architecture of T6SS clusters belonging to phylogenetic groups 1.1, 1.2a, 1.2b, 2, 3, 4A, 4B1 and 4B2 present in phytobacteria strains. The colour code of the genes correlates with the colour code shown in Fig. 1. PA14 indicates *P. aeruginosa* PA14; KT2440 indicates *P. putida* KT2440; Ech586 indicates *D. dadantii* Ech586; W619 refers to *P. putida* W619; GMI1000 refers to *R. solanacearum* GMI1000 and C58 to *A. tumefaciens* C58. *hcp* and *vgrG* genes vary enormously among species, when these genes are found within the clusters in the represented strain, the brackets indicate the positions at which they are located.

It is noticeable that T6SSs from different clades have unique genetic architectures (Fig. 4). Despite these variations, some genetic elements, such as the *tssBC* genes encoding the sheath (Fig. 1), appeared linked in all the clusters. In general, the genes encoding the T6SS core components are organized within one or two putative adjacent operons. However, accessory components can also be found within these clusters, e.g., *tagF* (Fig. 4, group 3, 4B1, 5) (Boyer *et al*., 2009), and in some cases genes encoding components of unknown function might confer novel T6SS functionality, e.g., *tagX* (Fig. 4, group 4B1) (Bernal *et al*., 2017). Another level of variation, when considering different or distinct organisms, is the position and number of the *hcp* and *vgrG* genes which vary within and outside the main T6SS clusters. For simplicity, they are not represented in Fig. 4, which only displays the canonical genetic architecture for each clade.

In plant‐related bacteria, T6SS genes from group 1.1 are organized in a single putative operon from *tssA* to *tssM* (Fig. 4). Two genes encoding putative regulatory components, i.e., *sfa2* and *fha*, are consistently found between *clpV* and *tssJ* (Fig. 4). The *sfa2* gene encodes the σ^54^ (RpoN) activator Sfa2, which has been involved (together with RpoN) in the regulation of H2‐T6SS expression in *P. aeruginosa* (Sana *et al*., 2013). Conversely, Fha has been described as a T6SS phosphorylation substrate involved in the post‐translational regulation of different T6SSs including *P. aeruginosa* (Mougous *et al*., 2007) via the Ser/Thr protein kinase PpkA and the antagonist phosphatase PppA. In *A. tumefaciens*, PpkA phosphorylated TssL that consequently interacts with Fha (Lin *et al*., 2014). Genes encoding FHA domain containing proteins are found within T6SS clusters from groups 1, 3 and 5 (Fig. 4). In some organisms that contain clusters from group 1, such as *V. cholerae* (Fig. 4, group 1.2), Fha has been shown to be essential (Zheng *et al*., 2011) although in this group, the *fha* genes are not genetically linked to the *ppkA/pppA* genes and might not mediate post‐transcriptional regulation of the T6SS (Ho *et al*., 2014) through a phosphorylation/dephosphorylation system. Interestingly, the *pppA* gene is fused to *tagF* in *A. tumefaciens* (Fig. 4, group 5) and the fusion protein (TagF‐PppA) has been proposed to encode a repressor of the system (Lin *et al*., 2014). The *tagF* gene is also present in group 3 clusters including H1‐T6SS of *P. aeruginosa* but is not fused to *ppp*A, the latter and *ppkA* are present as individual genes (Mougous *et al*., 2007). The presence of *tagF* in group 4B clusters is more puzzling since no *pppA* or any of the related genes encoding the regulatory cascade is present (Bernal *et al*., 2017) and might indicate that TagF in *P. putida* regulates the system through a different regulatory pathway.

The genetic architecture of group 1.2 slightly differs from that of 1.1. The *tssBC* and *tssEFG* genes encoding the sheath and baseplate components respectively, are still clustered together as in group 1.1 (Fig. 4). Same holds true for *tssK*, encoding a baseplate component and *tssJL* encoding components of the membrane complex, in the order *tssJKL*. The conservation of these gene clusters could be an indication of a canonical organization between the membrane platform and how it connects to the baseplate, including an interaction between TssK and TssL, as proposed for *E. coli* (Zoued *et al*., 2013). This organization is mostly kept in any of the other groups except for group 5 lacking *tssJ* and in group 2 where *tssE* is separated from *tssFG* by *tssJ* (Fig. 4). On the contrary, *tssA*, baseplate and *tssM*, membrane complex, have a more variable position in the different clusters and might be linked as in group 1.2 or not as in 1.1 (Fig. 4). The genetic link between the baseplate component TssA and the membrane protein TssM is yet quite frequent (groups 1.2A, 1.2B, 2, 4A, 4B2) which might indicate that TssA and TssM are functionally connected and have co‐evolved. However, the fact that in some instances TssM‐like proteins have additional domains including peptidoglycan‐binding domains (TagL/TagP), may also be an explanation for the variation in the genetic organization (Fig. 4, groups 2, 4B) (Aschtgen *et al*., 2010; Bernal *et al*., 2017). Interestingly, a second *tssA* gene is present in this strain and some other groups (Fig. 4, groups 1.2, 2). In the case of *tssA*, the two genes seem quite distinct and are unlikely to result from a duplication event, but duplication of a gene encoding a core component is possible as found in *A. tumefaciens* with consecutives *tssC* genes in the single putative operon corresponding to group 5 T6SS. In *V. cholerae* and *Francisella novicida*, it has been established that TssB (VipA/IglA) and TssC (VipB/IglB) form a heterodimeric sheath protomer with 1:1 stoichiometry (Clemens *et al*., 2015; Kudryashev *et al*., 2015). It is, therefore, unclear what the implication of an additional *tssC* gene could be but it might indicate a different conformation and/or functionality of the group 5 sheath.

The *clpV* gene is absent in the *P. putida* group 1.2a cluster (Bernal *et al*., 2017), which is unique for the T6SS^i^ clusters but was reported before with *Francisella* T6SS^ii^ (Bröms *et al*., 2010) (Fig. 4). The essential role of ClpV has been discussed previously and its absence might indicate either that an alternative Clp homologue takes over, as shown for *Francisella* (Brodmann *et al*., 2017), or that in the ClpV absence the T6SS is still functional but less potent since a single cell might fire only once (Zheng *et al*., 2011).

In some cases, genes encoding accessory proteins that provide additional functions to the system are found within the T6SS clusters. For example, the presence of a gene encoding the protein TagJ which recruits the ClpV ATPase to the sheath (Lossi *et al*., 2012; Förster *et al*., 2014) is observed in group 3 and group 5 clusters (Fig. 4), among which the well characterized *P. aeruginosa* H1‐T6SS or the *A. tumefaciens* T6SS are found (Lin *et al*., 2013; Förster *et al*., 2014). The presence of *tagJ* in *P. aeruginosa* H1‐T6SS has been suggested to be associated with a slightly different mechanism for dismantling the sheaths (Förster *et al*., 2014). In other cases, T6SS accessory genes have been identified but their function is still entirely unknown. This is the case in subgroup 4B1, where the first gene of a single putative operon encodes a small protein with no identifiable homology using any available databases (Fig. 4). The *P. putida* KT2440 K1‐T6SS is the sole cluster from this group characterized to date (Bernal *et al*., 2017) and the gene named *tagX1* for type VI accessory gene X (unknown function) could be coined a hallmark for clusters from the subgroup 4B1 (Figs 3 and 4).

Although T6SSs from different clades have distinct genetic organizations, all T6SS clusters likely produce structurally‐similar secretion machines. The diverse genetic architecture among clusters from different phylogenetic families might reflect divergences in the evolution and most likely reveals particular ways to modulate the assembly/disassembly of these systems.

## Phytobacterial T6SSs involved in interbacterial competition

Although the initial T6SS studies in phytobacteria aimed at understanding the role of this secretion system in virulence, subsequent analyses have shown that the T6SS plays a major role in interbacterial competition in both commensal and disease‐causing phytobacteria. *P. syringae, A. tumefaciens* and *Pantoea ananatis* are considered extremely deleterious plant pathogens. *P. syringae* is found in a wide variety of agricultural environments with more than 50 different pathovars able to infect numerous plant species (e.g., tomato by *P. syringae* pv. *tomato* DC3000 and bean by *P. syringae* pv. *syringae* B728a). Likewise, *A. tumefaciens* produces tumorigenesis in different crops, including potato plants, and *P. ananatis* strains cause disease in a wide variety of economically important crops (i.e., cotton, rice, corn, onion, pineapple and melon). Analyses of T6SSs in these phytopathogens showed that the systems are primarily used to inject toxins into bacterial competitors, thus providing both intra‐ and interspecies competitive advantages (Haapalainen *et al*., 2012; Ma *et al*., 2014; Shyntum *et al*., 2015). Several T6SS effectors with DNase (Tde) and antibacterial activity have been identified in *A. tumefaciens* (Ma *et al*., 2014) while the effectors of *P. syringae* and *P. ananatis* strains are still unknown. It was also noted that T6SS‐dependent competition is more efficiently carried out by *A. tumefaciens*, *in planta*, where it can outcompete *P. aeruginosa*, than *in vitro*, where *A. tumefaciens* is wiped out by *P. aeruginosa* (Ma *et al*., 2014). This showed that the impact of T6SS can be niche‐dependent, and that *in vitro* observations do not always correlate with the *in vivo* situation. In *P. syringae* pv. *tomato* DC3000, the T6SS‐2 confers growth advantages not only over other bacteria (i.e., Enterobacteriaceae), but also against eukaryotic microbes commonly found in soil and plant surfaces such as yeast and amoebas (Haapalainen *et al*., 2012). This suggests that this phytobacterium produces T6SS effectors that are effective against both prokaryotic and eukaryotic cells, as described for many other bacteria (Russell *et al*., 2013).

The capacity of T6SS‐active phytobacteria to annihilate prokaryotic competitors has been also studied in non‐pathogenic strains, e.g., *P. fluorescens* (Gallique *et al*., 2017), *P. putida* (Bernal *et al*., 2017) and *P. taiwanensis* (Chen *et al*., 2016). While *P. fluorescens* T6SS‐effectors are unknown, bioinformatics analysis has revealed a battery of 10 putative effectors‐immunity pairs for *P. putida* KT2440 including nucleases, pore‐forming colicins and a NAD(P)(+) glycohydrolase (Bernal *et al*., 2017). Of these, the T6SS‐dependent secretion and the killing activity of the polymorphic toxin Tke2, has been proven, as well as the effective protection conferred by its cognate immunity protein Tki2 (Bernal *et al*., 2017).

Over the last years, it has become clear that the main role of T6SS in phytobacteria is interbacterial competition rather than host manipulation and this holds true for both commensal and pathogenic plant‐associated bacteria.

## Role of T6SS in plant diseases

Although the event of T6SS effectors directly injected into plant cells has not yet been demonstrated, there are some examples in which the absence of the T6SS leads to an observable decrease of bacterial virulence in plants (Nykyri *et al*., 2012; Shyntum *et al*., 2015).

*Pectobacterium atrosepticum* (previously *Erwinia carotovora* subsp. *atroseptica*), a pectolytic bacterium that produces soft rot in plants, is one of the first phytopathogens for which T6SS activity was linked to virulence. This bacterium contains a single T6SS, and proteomic analyses identified VgrG and Hcp proteins (Fig. 1) in the secretome of cells grown with host extracts (i.e., potato stem and tuber extracts) (Mattinen *et al*., 2007). A pathogenicity assay showed that mutants in *tssC* and *tssK* (Fig. 1) are attenuated for virulence (Liu *et al*., 2008). Likewise, the two T6SSs of the closely related *Pectobacterium wasabiae* species seem to be involved in virulence during potato infection (Nykyri *et al*., 2012). However, how exactly T6SS confers virulence to *Pectobacterium* sp remains unclear. It could be due to direct injection of effectors into plant cells or to a competitive advantage over the plant microbiota during plant colonization, which would indirectly affect virulence. However, none of these possibilities has been examined in these studies. Yet, it is useful to note that *P. wasabiae* contains an impressive eleven *hcp* and *vgrG* genes, which are genes commonly linked to toxins/effectors genes. It is, therefore, a reasonable hypothesis to suggest that this battery of effectors is mainly used to annihilate competitors. A compromised virulence phenotype due to T6SS mutation has also been observed in *P. ananatis*, in which case the mutant loses the capacity to cause disease in onion plants (Shyntum *et al*., 2015). In this study, the T6SS has been shown to have antibacterial activity against a number of Gram‐negative bacteria, mainly other phytopathogens usually thriving within the *P. ananatis* niche (Shyntum *et al*., 2015). Whether this antibacterial capacity provides a fitness advantage to *P. ananatis* during plant colonization has not been determined, and, therefore, the exact mechanism that links *P. ananatis* T6SS with plant disease also remains to be elucidated.

In *R. solanacearum*, a destructive plant‐pathogen with a wide range of plant hosts within the Solanaceae family, the virulence of a *tssB* mutant is reduced when compared with the wild‐type strain (Zhang *et al*., 2014). This mutation affects considerably the motility and biofilm formation of the bacterium (Table 1) (Zhang *et al*., 2014), which could indirectly affect virulence. T6SS has been linked with biofilm formation in other phytobacteria such as the pathogen *Acidovorax citrulli* (Tian *et al*., 2015) and the non‐pathogenic *P. fluorescens* MFE01 strain (Gallique *et al*., 2017). However, an *A. citrulli* T6SS mutant was not affected in virulence (Tian *et al*., 2015). A possible link between T6SS and biofilm formation has also been described in animal pathogens. For example, a *P. aeruginosa tssM* (Fig. 1) mutant forms biofilm more efficiently than the wild‐type strain (Lin *et al*., 2015). Conversely, in pathogenic *E. coli* the *tssM* mutation produces a loss of adhesion to epithelial cells and a defect in biofilm formation on abiotic surfaces (de Pace *et al*., 2011). The connection between T6SS and biofilm is unexplained and a direct link would likely require the T6SS‐dependent secretion of an adhesin involved in biofilm, which has been predicted although not experimentally demonstrated in case of *Yersinia* (Pukatzki *et al*., 2007). Alternatively, regulatory elements encoded within the T6SS clusters (Sana *et al*., 2013) might regulate biofilm formation as well as T6SS activity (e.g., sigma‐54), which could explain the biofilm defective phenotype observed in some T6SS mutants. Interestingly, in *Citrobacter rodentium*, a chaperone‐usher cluster involved in fimbriae assembly is embedded within the putative T6SS operon, which suggests a possible co‐production of pili and T6SS (Gueguen and Cascales, 2013). Thus, the presence of non‐T6SS‐related elements encoded within or near T6SS clusters is an important factor to take into account when analysing pleiotropic effects of T6SS mutations.

**Table 1:** Phytobacterial T6SSs described in this study.

Phytobacteria strains	Proposed Function of T6SS^a^	References
Phytopathogens		
*Acidovorax citrulli*	Biofilm formation	(Tian *et al*., 2015)
*Agrobacterium tumefaciens*	Interbacterial competition and virulence	(Wu *et al*., 2008; Ma *et al*., 2014)
*Burkholderia thailandensis*	Interbacterial competition and virulence	(Schwarz *et al*., 2010)
*Dickeya dadantii*	Interbacterial competition	(Koskiniemi *et al*., 2013)
*Pantoea ananatis*	Interbacterial competition and virulence	(Shyntum *et al*., 2015)
*Pectobacterium atrosepticum*	Virulence	(Mattinen *et al*., 2007; Liu *et al*., 2008)
*Pectobacterium wasabiae*	Virulence	(Nykyri *et al*., 2012)
*Pseudomonas syringae*	Interbacterial competition and virulence	(Records and Gross, 2010; Haapalainen *et al*., 2012)
*Ralstonia solanacearum*	Biofilm formation	(Zhang *et al*., 2014)
Beneficial Bacteria		
*Pseudomonas fluorescens*	Interbacterial competition (biocontrol) and biofilm formation	(Decoin *et al*., 2014; Gallique *et al*., 2017)
*Pseudomonas putida*	Interbacterial competition (biocontrol)	(Bernal *et al*., 2017)
*Pseudomonas taiwanensis*	Interbacterial competition (biocontrol)	(Chen *et al*., 2016)
*Rhizobium leguminosarum*	Virulence	(Bladergroen *et al*., 2003)

aBased on the phenotype of T6SS‐mutants.

## T6SS and biocontrol

Besides *Rhizobium*, the other beneficial phytobacterial group in which T6SS has been analysed are strains of the genus *Pseudomonas*. Members of this genus are saprophytic bacteria that are widespread in the environment (Palleroni, 2010). Apart from the pathogens *P. syringae*, *P. savastanoi* and *P. aeruginosa*, the genus includes several strains of the rhizosphere species *P. putida*, *P. fluorescens* and *P. protegens* that can antagonize growth of plant pathogenic fungi and bacteria, and have been proposed as important biocontrol agents (Haas and Défago, 2005). T6SS is consistently present in this genus (Figs 2 and 3) and it is thus likely that this secretion system has an important function in *Pseudomonas* physiology and fitness. Importantly, a role for the T6SS in the biocontrol properties of bacteria has been established using *Pseudomonas* strains as models. For instance, *P. fluorescens* Pf29Arp, a strain that contains four complete T6SSs gene clusters and a total of nine *vgrG* genes (Marchi *et al*., 2013), can protect wheat roots from the disease caused by the pathogenic fungus *Gaeumannomyces graminis* var. *tritici*, and, although T6SS‐mediated protection has not yet been established, expression of T6SS genes was observed to be higher on fungus‐infected roots than in healthy roots (Marchi *et al*., 2013). *P. fluorescens* MFE01 contains a single T6SS that has antibacterial activity against a wide range of bacterial competitors, including rhizobacteria and clinical strains (Decoin *et al*., 2014). This strain is able to protect potato tubers against the soft‐rot disease caused by the phytopathogen *P. atrosepticum*, while a T6SS mutant (*tssC*) is not, and thus a role for T6SS in the PGPR properties of this bacterium was proposed (Decoin *et al*., 2014).

Biocontrol properties have also been detected in the soil bacterium *P. taiwanensis*, which has strong antagonistic activity againts *Xanthomonas oryzae* pv. *oryzae*, one of the most destructive pathogens of rice (Chen *et al*., 2016). Genome‐wide random mutagenesis allowed identification of the siderophore pyoverdine as the factor responsible of the growth inhibition of *X. oryzae* pv. *oryzae* by *P. taiwanensis*, and, surprisingly, the T6SS as the system required for full secretion of this siderophore (Chen *et al*., 2016). Pyoverdines are a group of peptidic siderophores produced and secreted by fluorescent *Pseudomonas* to scavenge iron from the environment (Visca *et al*., 2007; Cézard *et al*., 2015). While pyoverdine synthesis and recapture after iron chelation in the environment are well‐known processes, how the siderophore is exported outside the cell is still not clear (Visca *et al*., 2007). Since pyoverdine accumulates in the periplasm of *P. taiwanensis* T6SS mutants, authors suggest that this putative T6SS substrate can be collected from the periplasm (Chen *et al*., 2016). These features heavily challenge the actual T6SS mechanistic concept that involves secretion of protein compounds from the cytosol to the exterior of the cell. Nevertheless, direct secretion of pyoverdine via T6SS remains to be demonstrated, since the possibility that the T6SS acts only as a signal in the regulation of pyoverdine secretion cannot be excluded (Chen *et al*., 2016).

The link between T6SS and biocontrol has also been highlighted in a recent study using the biocontrol agent *P. putida* KT2440 (Bernal *et al*., 2017), a strain able to colonize the root of crop plants thus providing plant growth benefits (Espinosa‐Urgel *et al*., 2000; Molina *et al*., 2000). *P. putida* KT2440 contains three T6SSs and an impressive battery of T6SS effectors that are used by the bacterium to eradicate a broad range of competitors (Bernal *et al*., 2017). Remarkably, KT2440 has the ability to outcompete several dreadful phytopathogens, including *Xanthomonas campestris*, *P. syringae*, *A. tumefaciens* and *Pectobacterium carotovorum*, a capacity that is largely contributed by the T6SS (Bernal *et al*., 2017). This competitive advantage of *P. putida* KT2440 over phytopathogens also occurs *in vivo*. Indeed, the wild‐type strain can efficiently protect the leaves of *Nicotiana benthamiana* from deleterious necrosis inflicted by *X. campestris*, while a KT2440 T6SS mutant cannot (Bernal *et al*., 2017). Therefore, the T6SSs of KT2440, in particular, and of biocontrol bacteria in general, seems to be a primary mechanism to protect plants from the attack of phytopathogens, a function that has opened interesting prospects for biocontrol applications (Bernal *et al*., 2017).

## Concluding remarks and future perspectives

Whereas the term T6SS was coined following its discovery in *V. cholerae*, the pioneer work on *R. leguminosarum* suggested it was a system widely spread in pathogenic, symbiotic or commensal Gram‐negative bacteria. In fact, this is not different as compared to other secretion systems, which are often highlighted for their role in virulence but effectively are systems involved in communication with the extracellular environment and with other organisms. Here we have compiled all the knowledge available in phytobacterial T6SSs and drew a number of conclusions that would apply to any T6SSs. In brief, there does not seem to be a specialization of the T6SS machine for a given function or a bacterial lifestyle, despite an obvious variation in the composition and organization of T6SS gene clusters. These are grouped in no less than five distinct families but is for now reflecting an evolutionary drift of the system, which is mainly acquired through horizontal gene transfer rather than an adaptation to an ecological niche. This may finally happen, but for now it seems obvious that the specialization does not come from the system itself but from the toxins and effectors it secretes. In other words, one would not recognize a T6SS dedicated to the injection of eukaryotic or prokaryotic cells, and in many cases one can observe that one single system could do both, such as in *V. cholerae*. In contrast to animal pathogens, no T6SS effectors have yet been identified that could manipulate plant cells directly, and that may be one direction which would need to be investigated more thoroughly in the future. At the moment, all the phenotypic evidences collected from attenuated phytopathogen T6SS mutants cannot exclude that the T6SS defect results from a loss of fitness during colonization and the inability for the pathogen to compete with the rhizosphere residents. In this respect, and as much as we are hopeful that ‘probiotic‐like’ T6SS organisms would help manipulate the gut microbiota in humans and protect from pathogenic intrusion, the plant field is looking forward to the selection and engineering of T6SS super‐heroes that would wipe out phytopathogens, control agricultural loss, and make the public aware of the importance of microbes in this world.
